# Physical Testing in Sports Rehabilitation: Implications on a Potential Return to Sport

**DOI:** 10.1016/j.asmr.2021.09.034

**Published:** 2022-01-28

**Authors:** Mohamad Y. Fares, Hussein H. Khachfe, Hamza A. Salhab, Ahmad Bdeir, Jawad Fares, Hasan Baydoun

**Affiliations:** a College of Medical, Veterinary, and Life Sciences, University of Glasgow, Glasgow, Scotland, UK; b Department of General Surgery, University of Pittsburgh Medical Center, Pittsburgh, Pennsylvania, U.S.A.; c National Hospital for Neurology and Neurosurgery, University College London, London, UK; d Information Systems and Machine Learning Lab, Universität Hildesheim, Hildesheim, Germany; e Department of Neurological Surgery, Feinberg School of Medicine, Northwestern University, Chicago, Illinois, U.S.A.; f Department of Orthopedics, Mubadala Healthpoint, Sheikh Zayed Sports City, Abu Dhabi, United Arab Emirates

## Abstract

Strength and power constitute vital predictors for an individual’s quality of life and athletic performance. Measurement of these two parameters is very important in the world of sports science and medicine and necessitates a high level of accuracy and reliability. Several tests are used to measure strength and power, including the isometric maximal voluntary contraction test, the 1-repetition maximum test, and the Wingate test, as well as other tests that target upper and lower limbs. The unique characteristics present in each of these tests entail a subsequently unique mode of application during the process of rehabilitation. This helps athletic trainers and medical personnel evaluate recovery and decide on a potential return to sport. A comprehensive holistic approach that includes multiple testing, psychosocial assessment, and a gradual return to activity is best to achieve promising outcomes and preinjury athletic levels.

**Level of Evidence:**

V, expert opinion

Muscular strength, power, and function are vital predictors for the functional capacity of an individual.^1^ Maximizing these parameters contributes to a greater level of athletic performance and a higher quality of life, both physiologically and psychologically.^2^^,^^3^ Quantifying strength and power depends on the maximal force developed by the muscles involved, the rate at which the force developed, and neuromuscular coordination between different body segments.^4^

Rehabilitation in sports medicine is a process that aims to return the competing athlete to the sport as quickly and safely as possible.^5^ To do so, it is of pivotal importance to be able to measure and monitor muscular function, mobility, and strength throughout recovery.^5^ Accordingly, this necessitates the development of proper procedures that are able to quantify and assess these parameters for a complete evaluation of the physical fitness of the recovering athlete.^1^ Several tests are used to measure strength and power. These include the maximal isometric voluntary contraction test (MIVC), the 1-repetition maximum test (1-RM), the Wingate test, tests that target the upper limbs (upper quarter Y-balance test and seated medicine ball throw), and those that target the lower limbs (single hop test and jump tests).^1^, ^2^, ^3^, ^4^, ^5^, ^6^, ^7^ Each of these tests has strengths, limitations, and modes of application; as a result, each stands as a unique valuable tool in the world of sports medicine.

The MVIC test, which requires the individual to produce force on a fixed structure with maximal effort for 3 to 5 seconds, is often praised for its high reliability and objectivity.^1^^,^^8^^,^^9^ The 1-RM test is one of the best tests for assessing strength capacity in nonlaboratory environments and can do so for any part of the body.^10^ The Wingate test measures anaerobic capacity and power outputs through the concept of short-duration maximal efforts.^11^ Upper limb tests measure unilateral or bilateral upper limb mobility, stability, and strength. Finally, lower limb tests mimic movements encountered in many sports and offer simple and highly efficient ways of assessing lower body imbalances, power, and explosiveness.^12^ Using specialized equipment, these tests can help quantify peak force, relative force, rate of performance development, and other important parameters.

The use of these strength tests entails many applications in rehabilitation medicine and can provide reliable predictors for appropriate return to sport. Many sports use different tests to accurately assess the abilities of injured athletes and practitioners, which helps to prevent premature return to sport and provides adequate expectations of an athlete’s ability and performance. There are many strength and power tests specific to certain sports; however, for the scope of this review, we focus on some of the major tests used in rehabilitation medicine. These tests are reliable and imply benefits that can be reaped in many sports. As such, our aim is to explore their rationale, strengths, and limitations, and extrapolate their application in the world of sports rehabilitation.

## Return to Sport (RTS)

Allowing an injured athlete to RTS is one of the most challenging decisions in sports medicine. On the one hand, premature return can often predispose the athlete to injury recurrence and debilitating outcomes. On the other hand, sidelining athletes for too long can have negative consequences on fitness levels and postinjury performance. That is why the decision of RTS should be taken into meticulous consideration by the medical staff and physical therapy team.

The complexity behind such decisions led to the development of frameworks that can guide RTS. One very important framework is the StARRT framework, established by Ian Shrier in 2015.^13^ Shrier suggests 3 stages of thought in his framework that provide an assessment of the injured athlete.^13^ The first stage, health risk, evaluates tissue health and absorbable load. The second stage, activity risk, explores whether the tissue can withstand accumulative load and stress implicated by sport activities.^13^ The third stage, risk tolerance, provides risk modifiers and factors that can influence RTS; these include timing of injury in season, fear of litigation, and the use of painkillers.^13^ These frameworks can help guide medical teams through the rehabilitation processes and ensure proper, timely return. Nevertheless, to comply with such frameworks, reliable testing must take place to monitor the progression of the treatment.

Individual factors come into play when deciding whether an athlete should return to activity, including sex, age, previous injuries, type of sport, preinjury performance level, physical examination, rehabilitation, and injury characteristics.^13^^,^^14^ The main considerations behind RTS ensure that the athlete has no pain or significant limitations in mobility or stability, the injury has completely healed, and neuromuscular and proprioceptive function have recovered. As such, one of the most-used parameters to assess proper rehabilitation is symmetry between involved (injured) and uninvolved limb.^15^ The limb symmetry index (LSI), which is the ratio between the injured and the uninjured side, is often used by many experts to assess function and strength in an injured limb.^15^ An LSI of ≥90% is often considered an acceptable threshold for many injuries.^15^ Even though other important physical and mental factors come into play when deciding RTS, the LSI is considered a prominent marker that can be measured using many strength tests. Below we present some of the most notable strength and power tests that are often used in rehabilitation and assessing RTS.

### Maximal Isometric Voluntary Contraction Test

The MVIC test is regarded by many to be the gold standard of strength tests.^8^^,^^9^ An isometric contraction is a static form of exercise in which a muscle produces force without visible joint motion or substantial change in the length of the muscle.^16^ This test requires the individual to pull or push on a fixed object with maximal force for a short duration of time while a strain gauge measures the force executed. When performed with the assistance of a force plate, the MVIC can then measure maximal force, relative force, time to achieve maximal force, and rate of force development. When combined with jump tests, data from MVIC can offer valuable information such as the dynamic strength index.^17^ This parameter can measure the difference between an athlete’s maximal and explosive strength capacity.^18^ Khamoui et al.^17^ studied dynamic strength and suggested that explosive isometric force production within a short duration of time can correlate with vertical jump height. Although the study did have several limitations, the results may indicate that isometric abilities have velocity and time characteristics that can be transferred to sporting movements.

The MVIC test is often praised for its time efficiency and safety; it takes only minutes to complete and does not require the patient to lift heavy weights or exert a sustained amount of effort. In the setting of RTS and rehabilitation, it provides multiple benefits and can be safely administered to vulnerable populations such as injured personnel, without the fatigue associated with multiple repetitions.^19^ The MVIC has been used as a performance assessment tool for multiple sports. In weightlifting, for example, by adjusting the angle and body position, the MVIC test can assess the performance and function of any muscle in the body, a feature other tests do not have.^20^, ^21^, ^22^ Stone et al.^22^ explored the isometric mid-thigh pull, a variant of the MVIC test, and described how it can provide ample information on neuromuscular function, training alterations, and sport performance injury risk in weightlifters, and thus help evaluate recovery and predict RTS for these athletes. The MVIC has also been applied in American football injuries. A study by Beischer et al.^15^ explored RTS among young footballers with anterior cruciate ligament (ACL) injuries. The authors used isometric tests of quadriceps and hamstring strengths to calculate the LSI between the injured and the uninjured side.^15^ Using these calculations, the authors concluded that achieving muscle symmetry after ACL injury was not associated with recurrence, information that can help predict RTS in future patients.^15^ The high applicability of the MVIC test, evident by its safety, versatility, and practicality, allows it to be used in multiple sports. In addition, the ability to tackle different muscles allows it to assess function in both upper and lower limb injury settings.

Nevertheless, MVIC has its shortcomings. In many cases, the outcomes of research studies were specific to the equipment used in the corresponding laboratory and were not transferable to other methods and testing equipment.^23^ In addition, the MVIC test is specific to the angle used by the athlete, presenting limitations in assessing overall body strength.^24^ Moreover, a participant who has had previous surgery or complains of joint pain, instability, or edema may present with limited range of motion and may, hence, be unsuitable for this test.^24^ Finally, even though the test is relatively rapid to perform, setting up for it may be time-consuming, and the equipment needed to perform the test is not readily accessible to everyone.^25^

### One Repetition Max Test

The 1-RM test is a reliable tool commonly used to assess strength capacities, strength imbalances, and the effectiveness of training programs.^26^ To perform the 1-RM test, the athlete is asked to complete 1 repetition of an exercise in proper technique. The weight lifted is increased progressively until the athlete can no longer complete the lift with normal form. The maximal weight lifted with correct technique is considered the 1-RM.^27^

**Fig 1:**
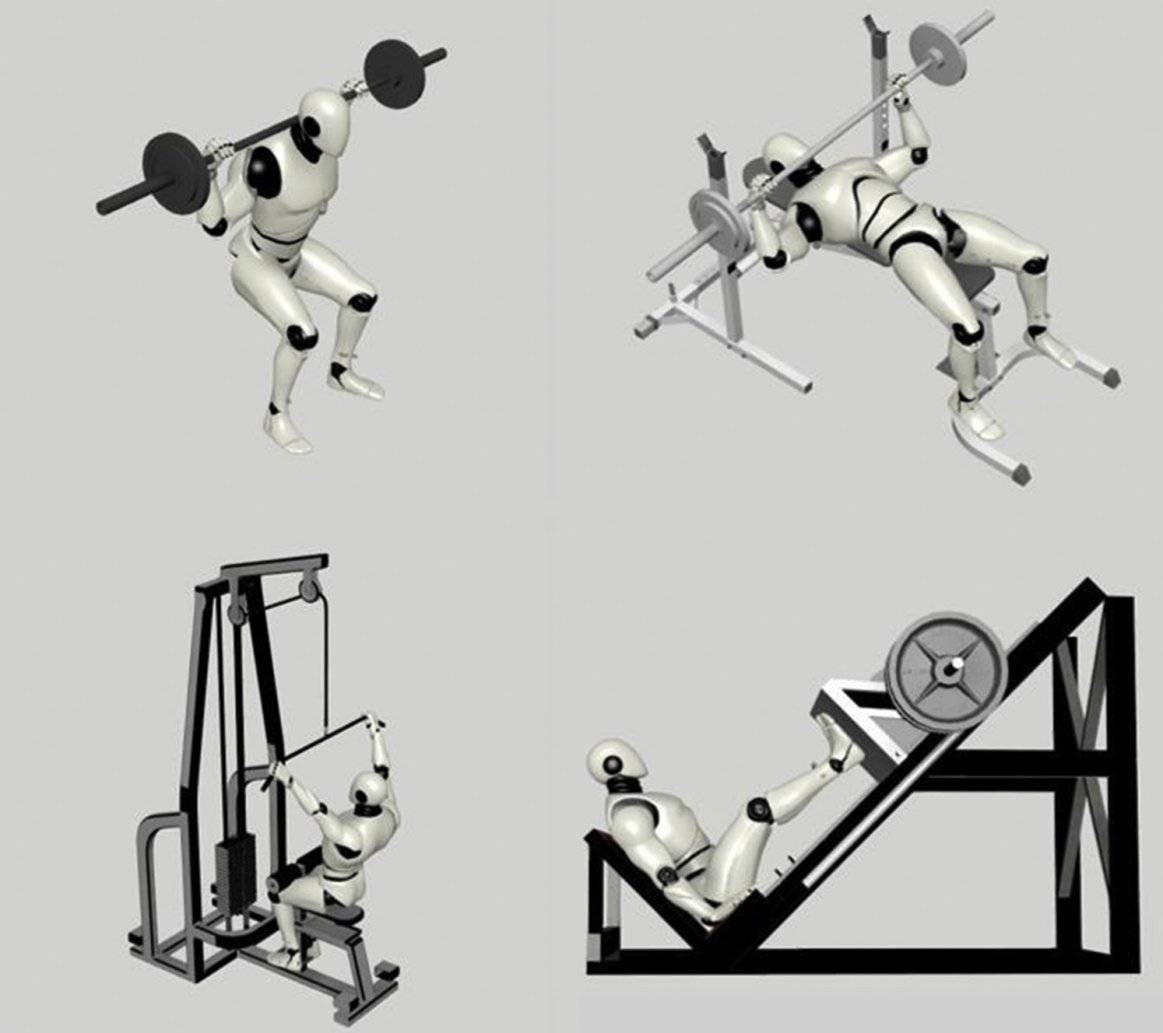
Different variations of the 1-RM test. Many reliable exercises can be used in the 1-RM test, including back squat, bench press, lat pull-down, leg press, and many others. The participant is required to complete 1 repetition of maximal weight lifted in proper form.

The 1-RM is popular in nonlaboratory settings, mainly owing to its easily accessible setup and its diversity.^10^ Many reliable exercises can be used in the 1-RM test, including back squat, leg press, bench press, lat pull-down, and others (Fig. 1).^27^^,^^28^ This diversity allows the 1-RM to be used in a variety of sports and to evaluate both upper and lower limb strength and power. Nevertheless, it is important to ensure that the exercise chosen is a reliable and safe predictor of 1-RM for the injured athlete, and given the relatively high rate of injuries during this test, its role in screening and regular monitoring would be superior to its role in rehabilitation and assessing RTS. The 1-RM test has always been debated with regard to safety and time consumption. It is often contended that it is unsafe for vulnerable individuals, especially compared with other tests.^27^^,^^29^^,^^30^ As a result, 1-RM can be used regularly as a reliable assessment of strength in healthy athletes rather than as a rehabilitation tool. In case of injury, 1-RM of recovering athletes can be compared to baseline to assess if return to preinjury level has been achieved.

### Wingate Test

**Fig 2:**
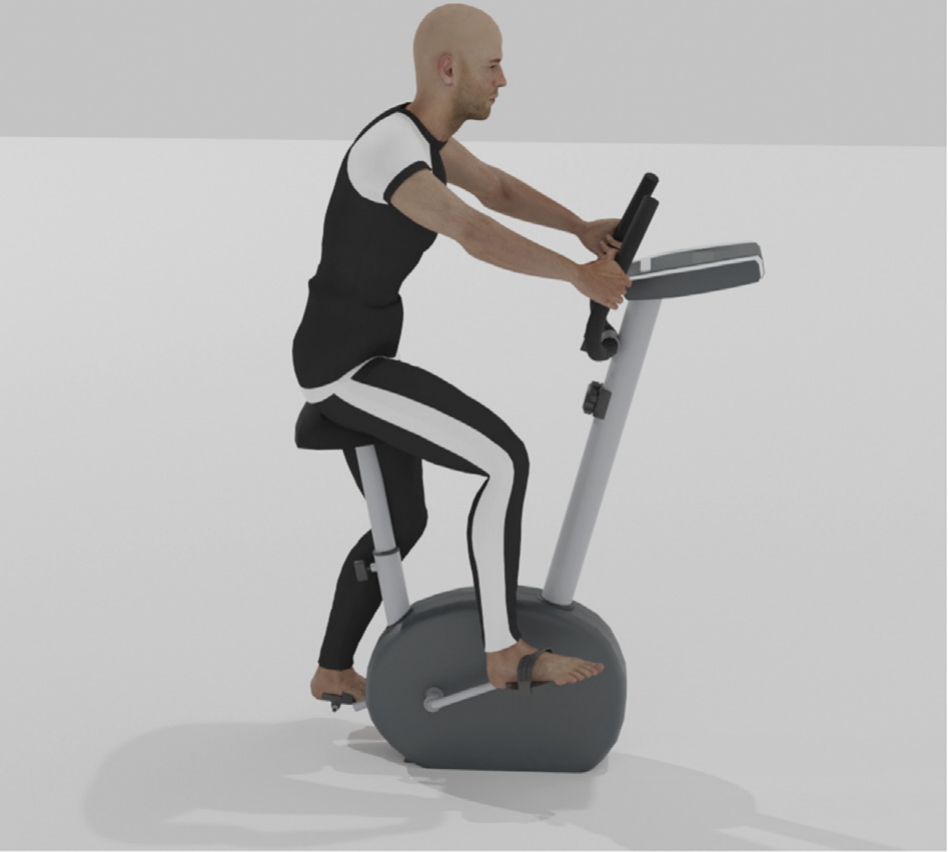
Execution of the Wingate tes. The participant is required to cycle on a cycle ergometer at a maximal effort for 30 seconds, usually against a resistance load. Using equations that integrate body weight, distance, and time, this test can provide valuable information relevant to the participant’s anaerobic strength.

The Wingate test, a fitness test developed in the 1970s, is one of the most famous laboratory fitness tests to assess anaerobic power outputs and capacity.^11^ These two parameters demand short-duration maximal efforts and are considered vital factors in sports and athletic performance.^11^ In the Wingate test, the individual is required to cycle on a cycle ergometer at a maximal effort for 30 seconds, usually against a resistance load (Fig. 2). The resistance load is administered a few seconds after the start of the test and is commonly equivalent to 7.5% of the participant’s weight, although load can be manipulated according to the requirements and capabilities of the presenting population.^31^^,^^32^

This simplicity and time-efficiency of this process is why the Wingate test is very popular worldwide. Using equations that integrate body weight, distance, and time, this test can provide measurements of peak power (PP), relative power output (RPP), anaerobic capacity (AC), and anaerobic fatigue (AF), a feature not present in other strength and power tests (Table 1). These measurements can provide valuable insight into the participant’s performance.^33^ PP, commonly observed during the first 5 seconds of the test, indicates the energy-generating capacity of the immediate energy system. RPP is equivalent to the PP relative to body mass. AC represents the total work accomplished during the test, and AF assesses the system’s total capability to produce adenosine triphosphate (ATP) via immediate and short-term energy systems.

**Table 1:** Relevant calculations conducted during the Wingate test to measure peak power (PP), relative peak power (RPP), anaerobic capacity (AC), and anaerobic fatigue (AF)

PP	force (kg) × distance (m) ÷ time (s)
RPP	PP (W) ÷ body weight (kg)
AC	[(PP – lowest power) ÷ PP] × 100
AF	Sum of each 5-second PP

The Wingate test has proven to be of particular use in sports that include short periods of maximal exertion, such as sprinting or cycling.^34^^,^^35^ Many injuries can sideline athletes for prolonged times, diminishing their anaerobic strength and decreasing performance. In these settings, the Wingate test can help assess anaerobic capacities of injured athletes and measure performance regularly throughout the rehabilitation process.^34^^,^^35^ RTS thresholds from Wingate tests can be inferred from preinjury performances or from the scores of a sample representative to that of the injured athlete. For example, a study by Emerson Franchini^36^ used a variant of the Wingate test to report expected performances from judo players according to weight divisions. After doing so, the author was able to classify the anaerobic strength of judokas using their Wingate test performances.^36^ Similarly, a study by Coppin et al.^37^ developed classification tables of Wingate test performances for the sports of American football and track and field. Using data from 77 collegiate athletes, the authors were able to report reference values for other athletes and trainers.^37^ This can provide immense benefit for predicting RTS in injured athletes and would prominently help guide rehabilitation efforts to optimize recovery.

Some may contend that the strong points of the Wingate test constitute its own limitations. The duration of the test is often considered too short to fully measure potential ATP turnover from the glycolytic-lactate system and, consequently, assess for anaerobic strength and power.^38^ In addition, as the test requires maximal exertion on a cycle ergometer, it may not be suitable for elderly individuals or patients with cardiac or respiratory problems.

## Upper Limb Strength Tests

### Upper Quarter Y-Balance Test

**Fig 3:**
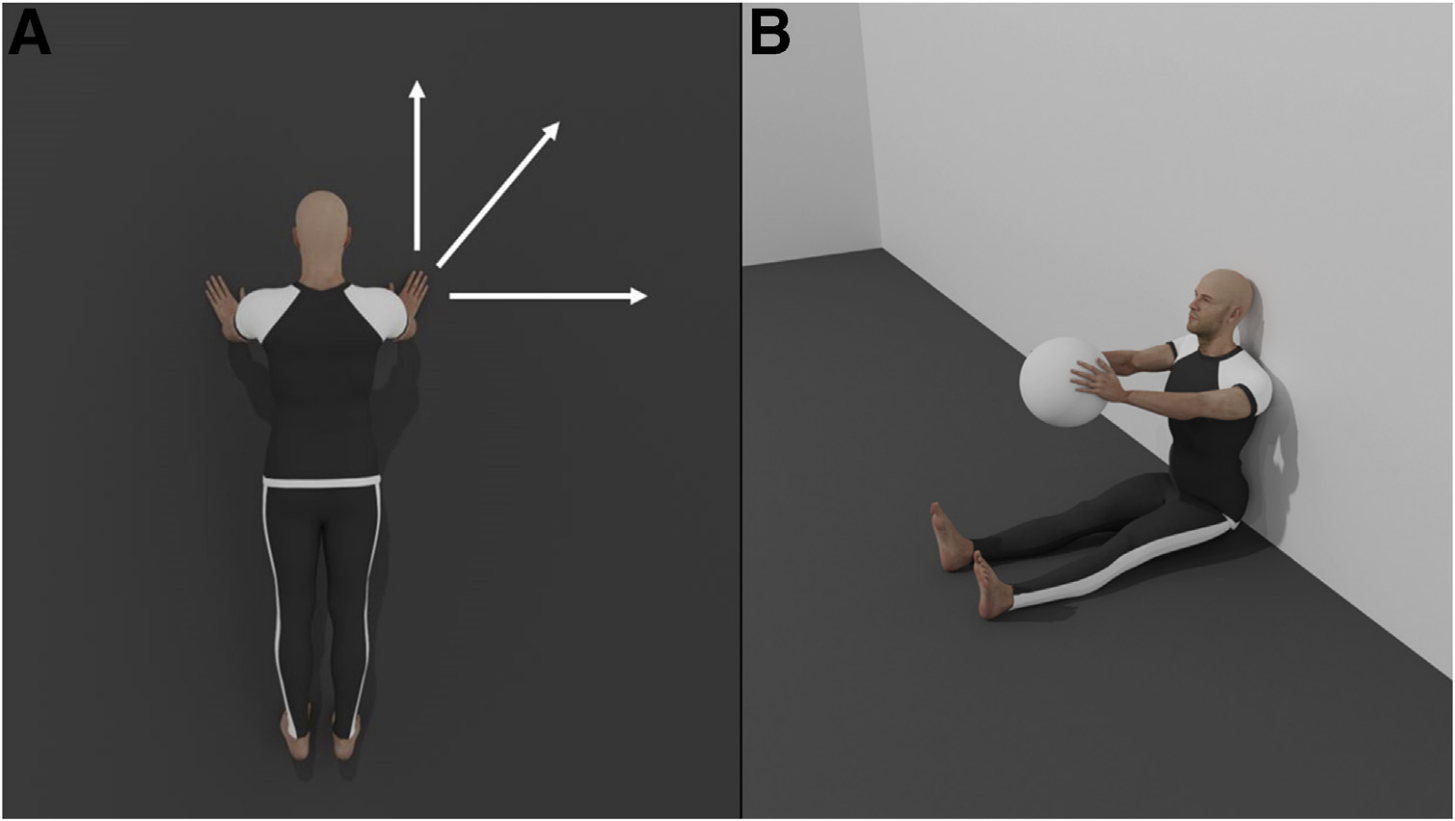
Upper limb tests. In the Upper Quarter Y-Balance Test (a), the participant is asked to keep their test hand still and have their free hand push a reach indicator into 3 different directions, while maintaining a pushup position. In the Seated Medicine Ball Throw (b), the participant is required to sit on the ground with their shoulders, back, and head pinned to the wall and their legs extended. They are then instructed to throw the ball straight ahead as far as possible without compromising their initial posture.

The upper quarter Y-balance test (UQ-YBT) is a screening tool to assess unilateral upper body function and mobility in a closed kinetic chain.^39^ It is usually performed by having participants adopt a pushup position, with feet placed shoulder-width apart (Fig. 3*a*).^40^^,^^41^ Participants are then asked to place their test hand on a stance platform and have their free hand push a reach indicator as much as possible in 3 different directions: medial, inferolateral, and superolateral (Fig. 3*a*).^40^^,^^41^ Participants then switch between the test hand and the free hand so that a bilateral measure can be provided for insight into upper limb strength symmetry.^40^^,^^41^ After a demonstration and a couple of trials, participants normally complete 3 trials under supervision to ensure validity and correct form.^40^^,^^41^ The evaluator then calculates the average distance for each direction and normalizes it according to upper limb length, providing a composite score that can be used to assess function, mobility, stability, and symmetry.

The UQ-YBT has proven to be very useful for assessing mobility and stability in unilateral upper limbs. Average distances reached by each upper limb can be compared to each other to assess symmetry and to reference values in the literature to evaluate mobility and function.^42^^,^^43^ This helps provide insight into the level of function reached by the recovering athlete. That being said, it is important to note some of the limitations imposed by this test. A study by Borms et al.^40^ explored the efficacy of UQ-YBT among other upper quadrant field tests and found that it did not relate to shoulder or elbow strength. The authors surmised the reason to be that strength assessment is performed in an open kinetic chain, and UQ-YBT is performed in a closed kinetic chain.^40^ In addition, the test is unsuitable for injured athletes with upper limb length discrepancy or restricted range of motion.^40^

### Seated Medicine Ball Throw

The seated medicine ball throw (SMBT) is a major test to assess bilateral upper limb strength and function.^6^^,^^40^ In this test, the participant is required to sit on the ground with shoulders, back, and head against the wall and legs extended (Fig. 3*b*), to isolate the upper extremities.^40^ The participant holds a medicine ball with elbows flexed and shoulders at 90° abduction (Fig. 3*b*).^40^ The participant is then instructed to throw the ball straight ahead as far as possible without the back, head, or shoulders losing contact with the wall.^40^ After a few practice trials, 4 supervised test trials are conducted, and the distance reached by the thrown ball is measured by tape. The average distance is used for analysis and assessment.^40^ Variants of the SMBT can also be used to assess unilateral upper limb function and strength, providing the ability to assess symmetry.^44^ In healthy, active participants, a 9% difference is expected between dominant and nondominant sides, and in the setting of rehabilitation medicine, this helps guide therapy and predict proper RTS.^45^

Many studies indicate a moderate to strong correlation between SMBT and upper limb power.^44^^,^^46^^,^^47^ Data derived from the SMBT can indicate bilateral and unilateral upper limb power, helping trainers and therapists assess function and symmetry in injured athletes and set goals and objectives.

## Lower Limb Tests

### Single Hop Test

**Fig 4:**
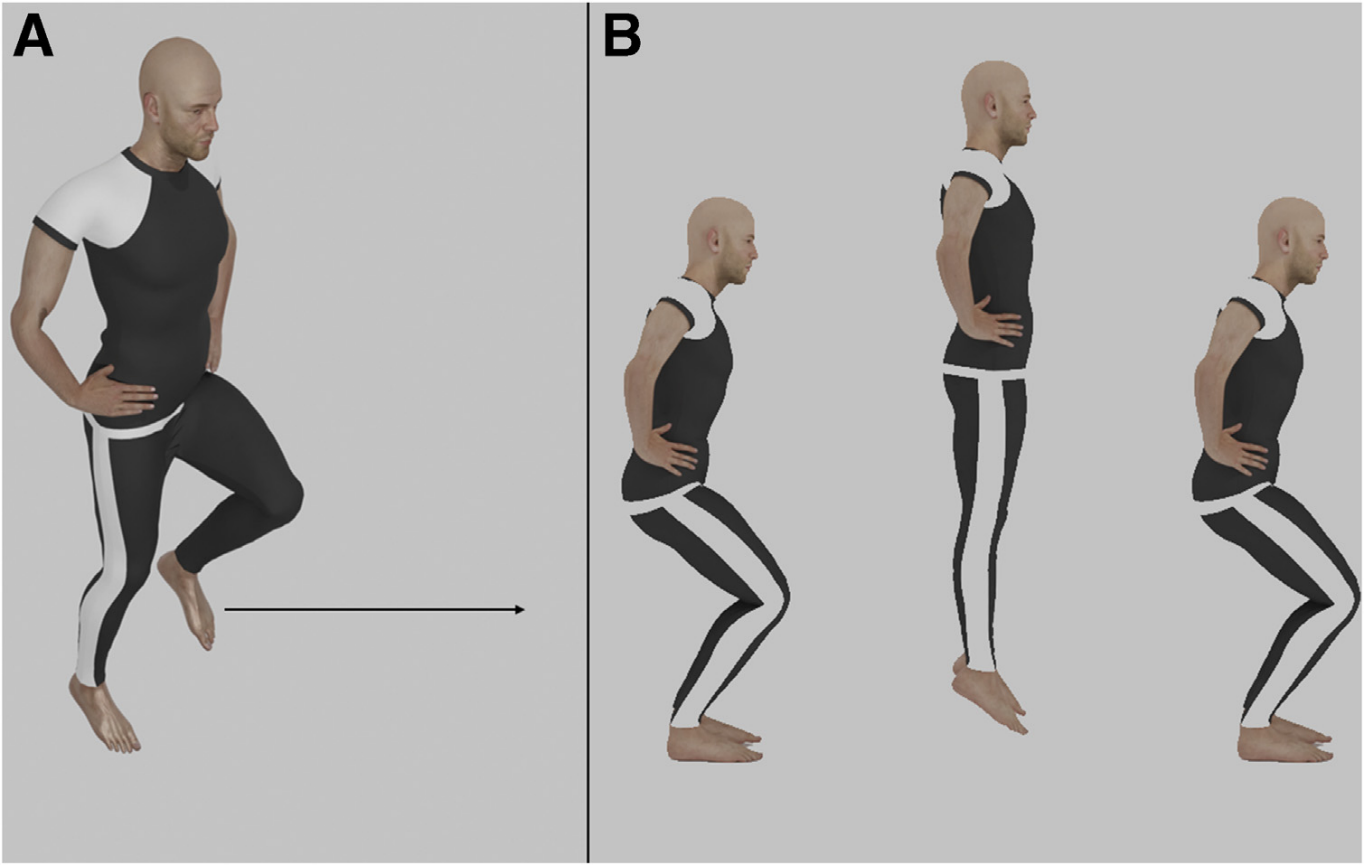
Lower limb tests. In the single hop test (a), the participant is asked to jump on a single leg as far as possible without compromising balance or landing. In a jump test (squat jump variant shown) (b), the participant is asked to jump on both legs as high and as forceful as possible without compromising proper landing or balance.

The single hop test is a simple and efficient way to test unilateral lower limb stability and function.^6^^,^^48^, ^49^, ^50^ To perform a single hop test, the participant is asked to jump on a single leg as far as possible, without compromising balance or landing (Fig. 4*a*).^39^ A supervisor then measures the distance from the start line to the heel of the landing leg and compares distances reached between the 2 lower limbs. As with other tests, the usual goal is to have a <10% difference in distance between the injured leg and the uninjured leg.^51^

This test has been used extensively in the literature, with a particular importance in ACL injuries.^48^, ^49^, ^50^, ^51^, ^52^ One study by Petschnig et al.^53^ explored the use of single hop test in surgical ACL patients and concluded that the single hop test can detect functional limitations in the lower limb up to 54 weeks after surgery. In these patients, the uninjured leg can be used as a control in evaluating RTS after ACL reconstruction.^53^ Nevertheless, recent evidence have shown that after ACL reconstruction, patients may still exhibit weaknesses in the injured knee despite achieving normal hop ratios.^54^ This was attributed to biomechanical impairments and deficiencies in proprioception and balance.^6^^,^^54^ As such, combining the single hop test with other lower limb tests may be necessary to achieve higher test sensitivity.

### Jump tests

Jump tests are simple and practical and are considered to be the most reliable tool to measure lower body power and explosiveness in athletes.^55^ For a jump test, a force platform records the force of the weight, jump time, and height. Although jump tests can also be conducted using contact mats, infrared platforms, and accelerometers, force platforms provide the best results with respect to accuracy and reliability.^56^, ^57^, ^58^

Jump tests include many variations; that being said, in all jump tests, the participant is asked to jump on both legs as high and as forcefully as possible without compromising proper landing or balance (Fig. 4*b*). Variations (starting position, involvement of arm swing, use of loaded weights) can help increase possible applications of these tests in multiple sports and can help derive formulas and calculate different parameters.^56^, ^57^, ^58^, ^59^, ^60^, ^61^, ^62^, ^63^, ^64^ Accordingly, they are often considered some of the most reliable tests for measuring lower body power and explosiveness—an important feature, since performance in most sports depends on the ability to quickly produce force.^65^

Jump tests have witnessed a surge in popularity among researchers and physical trainers worldwide.^66^ In the setting of rehabilitation medicine, jump tests can help monitor treatment progression in injured athletes, especially in sports that involve explosive lower limb movements such as jumping (basketball, volleyball, etc.).^67^ This can be done by studying the biomechanics of the jumping action by the athlete, evaluating any imbalances or muscular deficiencies and comparing the action to preinjury performance.^68^ However, several studies have highlighted the particular importance of jump tests in screening athletes for the purpose of injury prevention, rather than solely rehabilitation.^69^, ^70^, ^71^, ^72^ Hewett et al.^72^ recommended that in sports with a high risk of lower limb injuries, athletes should demonstrate high proficiency in motions such as jumping and cutting. These jump tests can help detect biomechanical faults in take-off and landing among healthy athletes and identify any muscle imbalances that may lead to injury.^69^

One must take into account a few considerations when dealing with jump tests. Varying take-off and landing positions, submaximal efforts conducted by the athlete, and improper flexion of the body joints before initiation can increase the chance of error in this test. Moreover, although the force mat is the most reliable tool to be used in jump tests, it is not very available and is not found in an ordinary setting.^57^

## Recommendations

Each of the tests described has its own advantages and limitations. These tests offer a reliable measure of the strength, function, mobility, and stability of an injured athlete’s body part, can help guide the rehabilitation process, and can predict potential RTS. Nevertheless, it is important to note that achieving acceptable scores on these tests does not necessitate full recovery or full ability to return to prior activity. A comprehensive individualized approach should be taken when dealing with athletic injuries and RTS.

Using multiple tests throughout the rehabilitation process can be necessary to ensure recovery and increase test sensitivity. This can help increase awareness with regard to injury recovery and would give better insight as to the level of function possessed by the injured athlete. For example, some athletes have persistent functional limitations with their injured lower limb despite achieving normal hop ratios, and the single hop test will have to be used in conjunction with other variables for better reliability.^54^ In addition, using multiple tests allows the trainer/medical personnel to attain a holistic view of the athlete’s fitness level. For example, using both UQ-YMBT and SMBT for a shoulder injury helps assess the mobility, stability, and strength of the injured shoulder. Moreover, performing Wingate testing throughout the rehabilitation process provides useful insight into the athlete’s anaerobic capacity. As a result, using different methods of testing in rehabilitation is necessary to achieve more sensitive outcomes.

In addition, it is very important to differentiate between return to sport and return to full activity. Achieving good ratios/scores on the tests described above may signify return of function, mobility, or stability; however, it does not necessarily imply the ability to perform physical skills and activities related to the sport.^73^ The athletic trainer should allow the athlete to return at a gradual and safe pace. Achieving preinjury performance levels requires gradual increase in loads and competition, as early returns have often been met with injury recurrence.^15^ In that regard, it is pivotal to monitor athletes’ progression after physical recovery and to allow a permissible increase in load before expecting return to preinjury performance.

Finally, considerations beyond physical examination and scoring should be made when deciding potential RTS. Many athletes exhibit prominent psychosocial impairments due to the injuries sustained, and this can cause apprehension from activity and fear of reinjury.^74^ A full assessment of the athlete’s psychosocial health should be undertaken to ensure the athlete has the right mentality for RTS. All these factors should be highlighted during the rehabilitation process to achieve favorable outcomes and acceptable postinjury performance levels. Safe RTS and athletic participation continues to remain a delicate art that we are trying to scientifically quantify day by day.

## Conclusions

**Fig 5:**
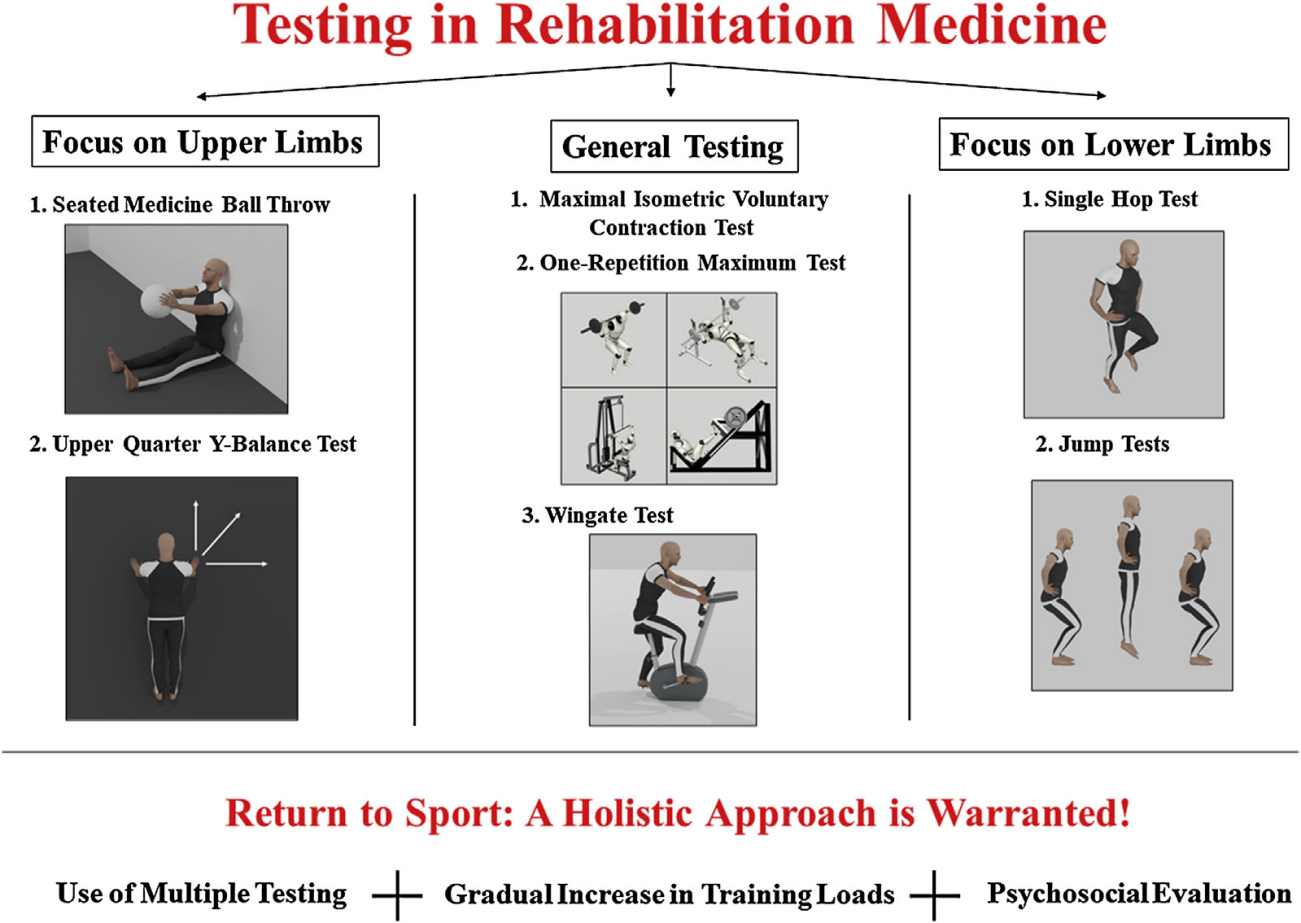
Testing in rehabilitation medicine and return to sport.

Muscular strength, power, and function are vital parameters that affect athletic performance and quality of life. In the world of rehabilitation medicine, assessment of an individual’s strength and power is very important and should be done with high accuracy and validity, as it allows for proper evaluation of recovery and timely prediction of RTS (Fig. 5). Several tests exist to measure and assess strength and power, including MVIC test, 1-RM, Wingate test, tests that target the upper limbs (UQ-YMBT and SMBT), and those that target the lower limb (single hop test and jump test). Each of these tests holds its own set of strengths and limitations and, accordingly, can offer a unique perspective to athletic trainers and medical personnel. Recommendations that should be taken into consideration during injury rehabilitation include the use of multiple testing to improve test sensitivity and attain a more accurate evaluation of recovery, allowing a gradual increase in loads following physical recovery, and adoption of a holistic individualized approach when considering a potential return to sport.

## Supplementary Data

ICMJE author disclosure forms
